# Does Enoxaparin treatment have any effects on the placenta in women with unexplained histories of habitual abortion? A case control study

**DOI:** 10.1590/1516-3180.2019.0511.R1.16042020

**Published:** 2020-06-22

**Authors:** Ayse Zehra Ozdemir, Bulent Ayas, Adem Kocaman, Mesut Önal, Gülnur Döğenci, İdris Koçak

**Affiliations:** I MD. Assistant Professor, Department of Obstetrics and Gynecology, Ondokuz Mayıs Üniversitesi, Samsun, Turkey.; II PhD. Associate Professor, Department of Histology and Embryology, Ondokuz Mayıs Üniversitesi, Samsun, Turkey.; III MSc. Doctoral Student, Department of Histology and Embryology, Ondokuz Mayıs Üniversitesi, Samsun, Turkey.; IV MD. Assistant Professor, Department of Obstetrics and Gynecology, Ondokuz Mayıs Üniversitesi, Samsun, Turkey.; V MD. Doctoral Student, Department of Obstetrics and Gynecology, Ondokuz Mayıs Üniversitesi, Samsun, Turkey.; VI MD. Professor, Department of Obstetrics and Gynecology, Ondokuz Mayıs Üniversitesi, Samsun, Turkey.

**Keywords:** Abortion, induced, Chorionic villi, Enoxaparin, Heparin, low-molecular-weight, Apoptosis, Placenta, Livebirth rate, Trophoblast proliferation, Vascularization

## Abstract

**BACKGROUND:**

It is very common to offer low molecular weight heparin (LMWH) medications to women with unexplained habitual abortion, to increase the livebirth rate. Although no benefit from LMWH has been clearly demonstrated, examination of the effects of enoxaparin on placental structure is lacking.

**OBJECTIVE:**

To assess placental structural changes in pregnancies treated with enoxaparin, compared with controls.

**DESIGN AND SETTING:**

Case-control study in an obstetrics and gynecology unit of a tertiary-level university hospital in Turkey.

**METHODS:**

Forty patients who had had term pregnancies and live births but also histories of habitual abortion were recruited for this study. Placentas were sampled using a systematic random sampling method. Tissue samples were obtained, embedded and sectioned for routine histological analyses. Hematoxylin and eosin staining was used. Surface area and length estimates from placental components were evaluated by using Image J. Cell proliferation and apoptosis were also assessed via immunohistochemistry.

**RESULTS:**

There were no significant differences between the groups regarding maternal age, abortion rate, birth weight or gestational age. Comparison of the enoxaparin and control groups showed that there were no significant differences in terms of surface area and ratios of placental components. We found that Bcl-2 was generally expressed at high levels in the enoxaparin group, while there was no difference in terms of Ki-67 between the groups.

**CONCLUSIONS:**

This study demonstrates that enoxaparin did not show any significant effect on the placental structure of cases that had histories of habitual abortion.

## INTRODUCTION

Habitual abortion is defined as the loss of three or more consecutive pregnancies prior to 20 weeks of gestation or a fetus weighing less than 500 g.^1^ Approximately 1%-2% of all women are affected.^2^ The etiology of habitual abortion is multifactorial and may include genetic, anatomical, infectious, thrombotic, autoimmune and endocrine causes.^3^ Although there is no established therapy for treatment of this patient group, it has been reported in several studies that use of progesterone and low-dose aspirin may be effective.^4^^,^^5^^,^^6^ Antenatal genetic consultations and psychological support are very important. Even though most published reports in the literature have failed to show any beneficial effect from low molecular weight heparin (LMWH) in enhancing the livebirth rates among women with unexplained habitual abortion,^7^^,^^8^^,^^9^ some other studies have suggested that the use of LMWH is beneficial.^10^ The mechanisms through which enoxaparin might have an effect during pregnancy are unclear.

The development of the placenta, starting with invasion of fetal trophoblast cells into the decidua in the early phase of pregnancy, is very important for the continuation of pregnancy. This process involves division, migration and differentiation of many cells and creates a dense vascular network. The placenta has been evaluated in many studies on patients with habitual abortion and some pathological conditions have been thought to cause abortion.^11^

Apoptosis is seen more frequently in the placenta of patients with habitual abortion than in that of patients with spontaneous abortion.^12^ In addition, it has been shown that perivillous fibrin deposition, chronic villitis, chronic histiocytic intervillositis, and plasma cell deciduitis are higher in chromosomally normal habitual abortions than in spontaneous abortions.^13^^,^^14^^,^^15^ Previous studies have also shown that placental invasion disorder is high, rather than thrombosis, in patients with habitual abortion.^16^^,^^17^

## OBJECTIVE

The aim of this study was to investigate placental changes in pregnancies with and without enoxaparin treatment, among patients who reached term and delivered healthy newborns.

## METHODS

### Ethical approval

The study was approved by the ethics committee of the Ondokuz Mayıs University, Samsun, Turkey, under the number 2019/725 on September 25, 2019. All procedures performed in this study were in accordance with the ethical standards of the institutional and/or national research committee and with the 1964 Helsinki declaration and its later amendments or comparable ethical standards.

### Informed consent

An informed written consent was obtained from patients or their relatives after the purpose of the study had been fully explained.

### Study design

All placental samples were obtained with local ethics committee approval, and informed consent was obtained from each patient. All the participants recruited were pregnant women with a history of miscarriage and they delivered live babies in the third trimester of this pregnancy. Before the start of the experiment, the reason for habitual abortion was investigated and no uterus anomaly, genetic pathology or antiphospholipid syndrome was detected in any of the patients. Patients with maternal diabetes, hypertension, kidney and liver disease, preeclampsia, intrauterine growth restriction and gestational diabetes were excluded. Furthermore, patients with a history of abortion due to antiphospholipid syndrome, identified uterine anomaly or known genetic pathological condition were also excluded from the study.

Placental samples were collected between September and December 2019, from 40 cases with or without enoxaparin treatment between January and September 2019. Twenty patients with a history of abortion and who were taking enoxaparin (Clexane 4000 anti-Xa IU/0.4 ml; Sanofi Aventis) during pregnancy were included in the enoxaparin group. Twenty patients with a history of abortion and who did not use any medications and then delivered live births at the third trimester were included in the control group. None of the patients had heparin-related complications, such as bleeding or allergies. All placentas were collected at delivery for the histological examination. Age, parity, previous abortions and weight were all recorded in relation to these forty patients.

### Placental sampling

After removing blood coagula and membranes, all placental samples were immersed in buffered formalin at least 72 hours. Two tissue samples were collected from each placenta. The fetal side of each placenta, in relation to the umbilical cord, was located and the tissue was marked by cutting 3 cm into the tissue on the right and left sides of the insertion of the umbilical cord. Full-depth columns of placental tissue were sampled as previously described.^18^

The tissue samples were washed overnight under a tap, dehydrated through a graded ethanol series (50, 70, 90 and 100%) (Sigma-Aldrich, USA), cleared in xylene (Merck, Darmstadt, Germany) and embedded in paraffin wax blocks. These paraffin-embedded tissue blocks were cut at a nominal section thickness of 5 µm using a microtome (RM2125RT; Leica, Nussloch, Germany). The sections were then dewaxed, rehydrated and stained with hematoxylin and eosin.

Three sections from each placental block were randomly selected and twelve microscope fields per section were captured (20x objective) by means of systematic random sampling.^19^ A total of 72 fields were analyzed from each patient’s samples. Images were collected using an Olympus BH2 microscope (Olympus, Tokyo, Japan) coupled to an F10 CCD digital camera (Panasonic, Osaka, Japan), and using the Pinnacle imaging software (Studio MovieBox Plus 710, Netherlands). The images thus acquired were analyzed using the ImageJ (NIH Software, Bethesda, MD, USA).

### Microscopic analyses

To measure placental differences, we estimated the mean cross-sectional areas of the stem (primary villus), secondary and tertiary villi and blood vessels, together with measurements of villus vascularization (vessel area to villus area ratios). In addition to measuring villus vascularization, we also estimated the diameter and thickness of the arteries within the stem villi. The ratios between the villus surface areas were used to monitor placental growth. To estimate the intervillous spaces, the total surface area of all the villi was summed up and then subtracted from the total surface area. In addition, we assessed the mean area of fibrin deposits on the peripheral surface of the villi, along with the mean area of syncytial knots and the thickness of syncytial trophoblasts.

### Immunohistochemical analyses

The avidin-biotin-peroxidase method was used for immunostaining. To detect Ki-67 and Bcl-2 antigen staining, 5 µm sections of placental samples were mounted on poly-l-lysine-coated slides and were separately incubated in the following antibodies: Ki-67 (SP6) rabbit monoclonal antibody (RM-9106-S1; NeoMarkers, CA, USA; diluted 1:50 in PBS),^20^ overnight at 4 °C; mouse anti-human Bcl-2 monoclonal antibody (Oncogene, Boston, MA, USA; diluted 1:20 in PBS),^21^ overnight at 4 °C; and biotinylated goat anti-polyvalent secondary antibody (TP-125-BN; Thermo Scientific, Fremont, CA, USA), for 25 minutes at 25 °C. Sections were also incubated with the avidin-biotin-peroxidase complex (Vectastain Elite ABC kit; Vector, Burlingame, CA, USA; diluted 1:50 in PBS) for 45 minutes at 25 °C. Peroxidase activity was detected using 3-amino-9-ethylcarbazole (AEC) chromogen (TA-125-SA; Thermo Scientific, Fremont, CA, USA) and these sections were lightly counterstained in Mayer’s hematoxylin (Merck, Darmstadt, Germany).

Fifteen fields were selected randomly in regions with Ki-67 positive nuclei and were examined at 100x magnification. Immunoreactivity was scored in more than 500 cells in villus cytotrophoblasts and in villus stromal cells, fetal endothelial cells and cells in the chorionic and basal plates of the placenta, for each case. For semiquantitative analysis of immunoreactivity, H-scores were used in this study, as previously described.^22^

### Statistics

Means and standard errors of the means were calculated for each group. Data distribution was tested using the Shapiro-Wilk normality test. The independent-sample t-test was used to compare normally distributed data between the enoxaparin and control groups. The data were analyzed using the Statistical Package for the Social Sciences (SPSS), version 25.0 (SPSS Inc, Chicago, IL, USA). Significance was accepted at P < 0.05.

## RESULTS

The mean maternal age, abortion rate, birth weight and gestational age did not differ between the two groups (P > 0.05) (Table 1). Comparison of placental area between the enoxaparin and control groups showed that there was no overall statistical difference. There was no significant difference between any groups regarding intervillous space area (Table 2). Comparison of stem, secondary and terminal villus areas between the enoxaparin and control groups did not show any statistical difference (P > 0.05).

**Table 1.** Comparisons of the mothers’ demographic characteristics between the enoxaparin and control groups

	Enoxaparin group	Control group	P-value
Age	28.2 ± 5.7	29.9 ± 5.1	0.294
Abortion rate	2.8 ± 1.1	3.1 ± 1.1	0.283
Birth weight (g)	3253.9 ± 396.4	3241.9 ± 374.8	0.918
Gestational age (weeks)	37.8 ± 1.3	37.7 ± 1.3	0.775

**Table 2.** Surface area and ratios of placental components

	Enoxaparin group	Control group	P-value
Total villus surface area (µm²)	3420463.6 ± 316931.8	3311996.6 ± 314831.2	0.284
Intervillous space area (µm²)	1638872.7 ± 362628.8	1779039.4 ± 314831.2	0.200
Fibrotic area (µm²)	183088.9 ± 113711.5	167556.2 ± 113984.4	0.669
Stem villus surface area (µm²)	1381308.5 ± 415346.7	1313494.8 ± 458938.9	0.627
Stem villus/total villus surface area	0.27132168	0.25800148	
Secondary villus surface area (µm²)	766550.1 ± 277585.1	767964.9 ± 359767.6	0.989
Secondary villus/total villus surface area	0.15056859	0.15084648	
Tertiary villus surface area (µm²)	1089516.2 ± 369572.6	1046972.6 ± 301399.7	0.692
Tertiary villus/total villus surface area	0.21400677	0.20565021	
Syncytial trophoblast thickness (µm)	26.5 ± 3.5	28.3 ± 5.2	0.196
Syncytial knot surface area (µm²)	28311.8 ± 10335	23265.4 ± 10810.1	0.140

While there was no significant difference between the groups in terms of the area of placental vascularization, the area of the tertiary blood vessels in the women treated with enoxaparin was slightly smaller than that of the controls (Table 3). There was no significant difference in stem villus artery thickness and diameter between the enoxaparin group and the control group (P > 0.05). Syncytial trophoblast thickness was slightly decreased in the enoxaparin group, compared with controls but without any statistically significant difference. Syncytial knot areas were increased in the enoxaparin group, compared with the controls (Table 2).

**Table 3.** Surface area and ratios of blood vessels

	Enoxaparin group	Control group	P-value
Stem villus artery surface area (µm²)	137781.2 ± 106295.9	95878.2 ± 39147.7	0.106
Stem villus artery/stem villus surface area	0.10347815	0.08566299	
Secondary villus blood vessel surface area (µm²)	95942.1 ± 56797.6	114429.3 ± 77698.8	0.396
Secondary villus blood vessel/secondary villi surface area	0.12667177	0.11121584	
Tertiary villus blood vessel surface area (µm²)	198576.9 ± 126475.7	218740.5 ± 86191.5	0.559
Tertiary villus blood vessel/tertiary villus surface area	0.18101836	0.21258895	
Stem villus artery diameter (µm)	218.7 ± 87.7	195.4 ± 67.4	0.351
Stem villus arterial wall thickness (µm)	72.9 ± 27	68 ± 32.9	0.610

**Figure 1.**
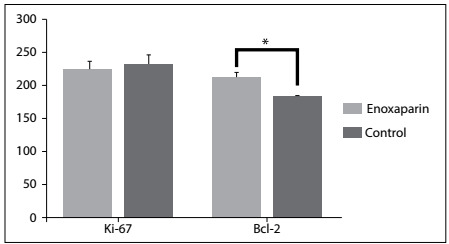
Average H-scores for Ki-67 and Bcl-2 in the enoxaparin and control groups. *Represents the statistical difference between the groups, P ≤ 0.01.

**Figure 2.**
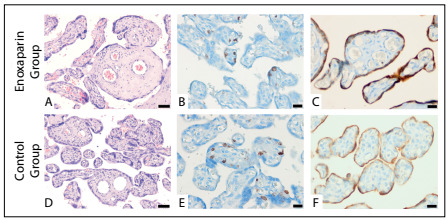
Stained sections from term placentas in the enoxaparin and control groups: hematoxylin-eosin (H&E) staining (A and D, obj. 10x) and immunoreactivity for Ki-67 (B and E, obj. 40x) and Bcl-2 (C and F, obj. 40x). Expression of Ki-67 and BCL-2 in normal-term villous cytotrophoblast tissue was viewed by means of rabbit monoclonal Ki-67 antibody and mouse anti-human Bcl-2 respectively. The specimens were counterstained with Mayer’s hematoxylin. Bar (A and D): 50 µm; bar (B, C, E and F): 20 µm.

Cell proliferation was assessed using the Ki-67 assay and was apparent in the nuclei of the villus trophoblasts in both groups. Immunoreactivity of syncytial trophoblasts was also found in the syncytial knots. Statistically, there were no significant differences between the groups in terms of Ki-67 immunoreactivity (P > 0.05) (Figure 1). Immunoreactive Bcl-2 protein was detected immunoenzymatically in all groups, to assess apoptosis. Immunopositive cells were located especially in the syncytial trophoblast cytoplasm of the intermediate and terminal villi and were seen as a strong, brown, cytoplasmic stain (Figure 2). In the enoxaparin group, the trophoblasts lining the villi exhibited positive cytoplasmic areas that were continuous with non-labeled cytoplasm. A significant increase in the mean number of Bcl-2 immunoreactive cells was found in the placental villi of the enoxaparin group, compared with the control group (P ≤ 0.01).

## DISCUSSION

The placenta is an important organ for the continuation of pregnancy. Morphometric investigation of the placenta is crucial for understanding the pathogenesis of disorders affecting the fetus. The placental villus area changes depending on the level of placental ischemia.^23^ The intervillous space decreases through invasion of syncytial trophoblasts, accumulation of fibrous tissue and syncytial degeneration due to deepithelization.^24^ Syncytial knots emerge through accumulation of syncytial nuclei, and the number of knots increases with rising malperfusion and hypoxia levels.^25^ Perivillous fibrin storage takes place in the eosinophilic fibrin conglomeration around the villi.^26^ The fibrin accumulation disrupts oxygenation around the villi and causes ischemic necrosis.^15^

We did not notice any differences regarding villus area, intervillous area, syncytial knot area and fibrotic area between the patients who underwent enoxaparin treatment and those who did not, in investigating the placentas belonging to pregnancies with term live births that were obtained from our patients with previous histories of unexplained habitual abortion. It had already been observed that the levels of placental perivillous fibrin, chronic villitis and plasma cell deciduitis were higher in patients with recurrent abortion who presented normal karyotypes, compared with patients with spontaneous abortion.^14^

It was also previously seen that there was more infarct, fibrin deposition, syncytial knot development and fibrosis in the placenta of patients with antiphospholipid antibody syndrome (APS) and antiphospholipid antibody-like syndromes (APS-like), compared with normal patients. Moreover, when patients with APS and APS-like syndromes who underwent either normal birth or abortion were compared, their placental histological findings were similar between the two populations.^27^ In comparing APS-positive and negative patients, patients with chromosome anomalies and patients in a control group, it was noted that there was no significant difference among the groups regarding intervillous thrombus.^17^

In another study, it was postulated that chronic histiocytic intervillositis was associated with recurrent abortion.^13^ In placental studies, it was noticed that placental trophoblast invasion disorder is more common than placental thrombosis among thrombophilic patients.^13^^,^^16^^,^^17^ In our study, it was found that the syncytial trophoblast thickness decreased but that the stem villus artery diameter was increased in the patients who underwent heparin treatment, compared with those who did not receive any treatment. However, these findings did not present any statistically significant difference.

Recent studies focusing on usage of low molecular weight heparin have suggested that there is insufficient evidence for application of this treatment among patients with unexplained habitual abortion.^7^ In a study conducted by Lu X et al., aspirin was given to the patient groups with habitual abortion and abnormal prenatal thrombocyte aggregation, while low molecular weight heparin was used for patients with high levels of d-dimer. It was seen that thrombocyte aggregation was lowered, and that the d-dimer level was also lowered throughout the period of pregnancy. A live birth rate of 89.2% was obtained in the group with unexplained recurrent spontaneous abortion.^10^

In a 2010 randomized controlled study, patients with unexplained habitual abortion were included in the study. One group received aspirin and heparin, one group received only aspirin and the third group received placebo. There was no statistically significant difference among the groups regarding the rate of live births. In a study by Doliztky et al., 50 of the patients with unexplained abortion received aspirin treatment and 54 of them received enoxaparin treatment. It was found that the live birth rates of the two groups were similar. Moreover, the placental doppler flow was also found to be similar among the groups.^8^ In a study conducted by Maged et al., on patients with unexplained habitual abortion, one group received low-dose aspirin (75 mg) and heparin (5000 IU) and the other group did not receive any medication. The evaluation criteria were that the first trimester was completed and that there was no statistically significant difference among the groups.^9^

During the development of the placenta, trophoblastic cells proliferate and the placenta becomes remodeled through apoptosis.^28^ Thus, apoptosis continues as a dynamic process in placental development. In this process, proapoptotic and antiapoptotic factors should be in balance.^29^ Bcl-2 is an antiapoptotic marker that inhibits apoptosis. Several studies have shown that antiapoptotic Bcl-2 levels are lower in the placenta of patients with habitual abortions than in the normal population.^12^^,^^29^^,^^30^

Furthermore, it has been indicated that Bcl-2 expression, which is low in the early weeks of normal pregnancy, increases during late weeks of pregnancy. This increase may provide continuity for the placenta.^31^ In addition, decreases in Bcl-2 levels are thought to be a process that reduces chorionic villus survivability.^30^ In our study, antiapoptotic Bcl-2 levels were found to be higher in the enoxaparin group than in the control group. This suggests that enoxaparin may decrease the effect of placental apoptosis.

However, in the current study, there was no difference between the groups regarding Ki-67 expression, which was measured to demonstrate placental proliferation. These results may be taken as fairly representative of what may be expected of the placenta at the end of full-term pregnancy, in comparison with the placenta at the early stage of pregnancy. Ki-67 expression has been shown to be relatively high in the placenta during early pregnancy but that it decreases over subsequent weeks.^32^ On the other hand, previous studies indicated that the profile of Ki-67 expression was lower in the placentas of patients with habitual abortion.^33^ Therefore, we believe that investigation of the effect of enoxaparin in placental samples taken in the early weeks of gestation may be more meaningful for evaluating the Ki-67 expression profile.

In our study, we did not notice any histomorphological alterations in placental histopathology due to enoxaparin usage, among the patients with term pregnancies. One of the limitations of our study was the absence of a group without habitual abortion. Because of this, no comparison could be made between a group without habitual abortion and a group with term pregnancy placenta. Moreover, the low number of patients included in our study was another limitation. However, it is important that the patient groups are comprised of individuals with idiopathic abortion that is investigated in terms of genetics, thrombophilia and APS.

## CONCLUSION

This study is important in that it investigates the effects of enoxaparin usage, which is a costly and difficult treatment, among patients with unexplained habitual abortion, in terms of placental morphology. However, for clearer understanding of this subject, prospective studies with larger populations are needed.
